# Cochlear Implantation in Vestibular Schwannoma Surgery: Diagnostic Accuracy Analysis of Intraoperative Monitoring with Intracochlear Electrode

**DOI:** 10.1097/MAO.0000000000004437

**Published:** 2025-02-21

**Authors:** Elisabetta Zanoletti, Stefano Concheri, Giulia Tealdo, Diego Cazzador, Valerio M. Di Pasquale Fiasca, Sebastiano Franchella, Giuseppe Impala’, Davide Brotto

**Affiliations:** ∗ Section of Otorhinolaryngology, Department of Neuroscience, University of Padova, Padua, Italy; † Unit of Otorhinolaryngology, Azienda Ospedale Università Padova, Padua, Italy

**Keywords:** Cochlear implant, Cochlear nerve monitoring, Intracochlear test electrode, Vestibular schwannoma

## Abstract

**Objective:**

To investigate the role of intraoperative cochlear nerve (CN) electric monitoring with MED-EL intracochlear test electrode (ITE) in assessing the CN functional integrity.

**Setting:**

Tertiary referral center.

**Patients:**

Patients with intrameatal or 2 to 13 mm in the cerebello-pontine angle vestibular schwannoma (VS), not suitable for hearing preservation surgery but eligible for tumor resection via translabyrinthine approach and simultaneous cochlear implant (CI) rehabilitation.

**Intervention:**

ITE was used to register electrically evoked auditory brainstem response (eABR) before and after VS resection. All patients with anatomical preservation of CN underwent CI, regardless of eABR results, which served as the index test and was compared with postoperative sound perception by CI stimuli (gold standard test).

**Results:**

Twelve of seventeen cases allowed anatomical preservation of CN and were considered for the study. Seven of twelve cases demonstrated sound detection with CI, and six of twelve showed some degree of speech discrimination. eABR test with ITE achieved an accuracy of 66.7%, a sensitivity of 42.9%, and a specificity of 100%. Positive and negative predictive values were 100% and 55.6%, respectively.

**Conclusion:**

When eABR can be evoked with ITE, the attempt of CI was likely to be successful, whereas in cases of eABR absence, other factors should be considered to reduce unsuccessful CI and not preclude rehabilitation in patients who would benefit from CI. Further studies and longer follow-up are needed to analyze the role of ITE in VS surgery with CI.

## INTRODUCTION

There is ongoing debate regarding the optimal treatment for small vestibular schwannoma (VS), with the majority of current evidence favoring observation (1,2). Nevertheless, it is widely acknowledged that despite the extensive literature on the topic, the reported level of evidence remains low (1) and no treatment approach showed an unequivocal superiority to others with a high level of evidence (2). The hearing outcomes of the three therapies—observation, radiotherapy, and surgery—should be evaluated in the long term. Hearing decline progresses inexorably over time (3–5) and recent trends seems to favor proactive surgery in small growing tumors, to maximize the outcomes of facial nerve and hearing preservation (6–9). Very small intrameatal tumors with excellent hearing at diagnosis may represent an exception to the inexorable hearing decline that typically occurs over time during observation (10).

Hearing preservation surgery (HPS) is theoretically feasible in any tumor with serviceable hearing, but the literature indicates that the best hearing outcomes are achieved when hearing is optimal at the time of diagnosis (7,11–15). Otherwise, observation remains the mainstay of treatment and in our opinion, when the favorable conditions to HPS are missing, active therapy (radiotherapy or surgery) can be considered only for growing tumors. The option of cochlear implant (CI) is used to rehabilitate postsurgical deafness, either after failed HPS, or when tumor growth with nonoptimal hearing would suggest surgical therapy. The attempt to surgically preserve a viable cochlear nerve (CN) has become the new goal and, consequently, the assessment of the functional integrity of the CN after tumor resection is of paramount importance.

CN “residual” function after translabyrinthine (TL) surgery has been recently investigated with intraoperative tests by various authors (16–19). The aim of these studies was to assess the predictive value of intraoperative CN stimulation technique and discriminate the cases where, although anatomically preserved, the CN had lost its fitness to CI. This aspect represents a key issue to optimize CI rehabilitation and maximize the cost–benefit burden. The electrically evoked auditory brainstem response (eABR) is the most frequently used technique for intraoperative monitoring (20), but its reliability in predicting the CI auditory outcomes is yet to be defined because of the still unsolved issue of false-negative cases (17–19).

The aim of the present study was to investigate the role of intraoperative CN electric monitoring with MED-EL intracochlear test electrode (ITE) to assess the CN functional integrity and the final auditory outcome of the CI.

## MATERIALS AND METHODS

**FIG. 1:**
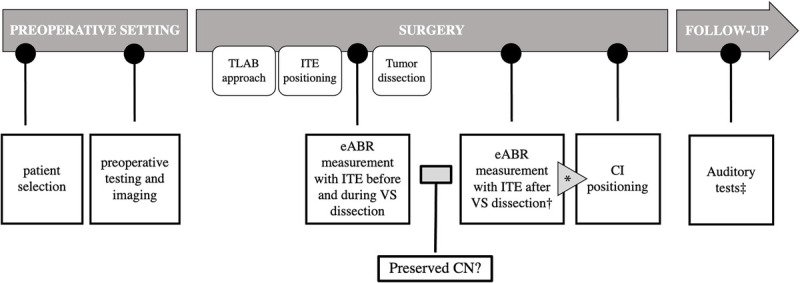
Outline of the study design. * All patients with an anatomically preserved nerve received CI placement, regardless of eABR responses. † eABR after tumor dissection was the index test of the study. ‡ Sound detection arising by CI stimulation was considered the reference test.

We retrospectively considered patients affected by small VS who were eligible for VS resection and simultaneous CI rehabilitation between November 2020 and July 2023 at a tertiary referral center. Intraoperative electric CN monitoring was performed with ITE by MED-EL, before and at the end of tumor resection (Fig. 1). CI was simultaneously positioned in all patients with anatomically preserved CN, regardless of the result of the intraoperative test.

### Patients’ Selection

The whole series of small VS in our institution was managed according to our institutional protocol (7,9,21). Cases with “good” hearing underwent observation or, in selected cases, HPS. Favorable candidates for HPS were those with an average pure tone audiometry (PTA) ≤30 dB, a word recognition score (WRS) ≥70% and normal or slightly altered ABR. All the other cases (PTA >30 dB, WRS < 70%, and massively altered or absent ABR) were observed. For growing tumors, early surgery with TL approach with the option of CI was offered.

The mentioned criteria were considered for candidacy to surgery and the following criteria were considered in the present study for patients’ selection.

Inclusion criteria were (i) patients underwent TL surgery for small (≤10 mm tumor size in the cerebello-pontine angle [CPA]) growing VS and unserviceable preoperative hearing (classes C–F according to the Tokyo classification) (22) or patients ineligible for HPS, or (ii) patient’s decision for upfront surgery regardless of tumor growth, with preoperative hearing ineligible for HPS. Exclusion criteria were conditions unsuitable for surgery or patient’s refusal. Both sporadic and neurofibromatosis type 2-associated VSs were included in the study.

Patients signed an informed consent, and each therapy option was explained and discussed.

CI was positioned only in those patients with anatomically preserved CN at the end of tumor resection (Fig. 1); the evaluation of CN preservation was assessed by the surgeon performing the procedure. This subgroup of patients represented the present series.

The study was conducted in accordance with the principles of the Declaration of Helsinki, Italian privacy and data laws, and in-house rules of our Otolaryngology Section. STARD 2015 was the reference guideline in the design of this study (23).

### Radiological Pre- and Postoperative Evaluation

Tumor dimension was measured with gadolinium-enhanced MRI-T1 sequences in its largest extrameatal size according to the Tokyo consensus meeting (22). Free fundal cap was evaluated in MRI-T2 cisternography sequences, and presence of cystic areas within the VS was determined in T2- and gadolinium-enhanced T1 sequences. A high-resolution bone CT scan was performed preoperatively to assess patient’s temporal bone anatomy, and after surgery to check CI placement.

MRI was scheduled at 1, 3, 5, and 10 years after surgery to rule out recurrences.

### Audiological Pre- and Postoperative Evaluation

Pre- and postoperative PTA was measured at 0.5, 1, 2, 4 kHz, according to Kanzaki et al. (22). Contralateral ear was masked with 65 dB masking noise when air conduction thresholds between the two sides differed more than 40 dB or bone conduction thresholds more than 5 dB. Speech tests were based on lists of words presented at increasing loudness; the final score (WRS) was reported as the maximum percentage of words correctly repeated by the patient, regardless of the intensity of the sound. Follow up was scheduled at 3, 6, 12, and 24 months after CI activation (1 mo after surgery); patients underwent a CI fitting session to optimize the CI performances and then repeated the audiological tests.

### Surgical Protocol

All procedures were performed by the same team of surgeons via TL approach. At the end of the approach, the round window was reached via posterior tympanotomy. ITE was fully inserted, and the reference electrode was placed under the temporalis muscle. Complete tumor removal started and was performed under direct CN stimulation by ITE. It was applied every 10 minutes or before and after any surgical maneuver that could put the CN at risk. At the end of tumor removal, the fundus of the internal auditory canal was explored with an endoscope to assess no residual, confirm the anatomical integrity of the CN, and confirm the microscopic intraoperative findings. Intraoperative test monitoring was then recorded. CI was placed regardless of eABR responses. The ITE was removed after tumor removal and replaced by the CI array (Fig. 1). The receiver/stimulator was housed in a subperiosteal pocket behind the ear beneath the temporalis muscle. MED-EL Flex28 was the implanted device. Correct CI positioning was intraoperatively checked with impedance and electrically evoked compound action potential (ECAP) assessment. Facial nerve was monitored during surgery with continuous electromyography (NIM-Response 3.0, Medtronic Xomed Inc.) and its function verified with a stimulation probe. Postoperative facial nerve status was graded according to the House–Brackmann scale (24).

### Intraoperative eABR Measurement

Intraoperative CN integrity was assessed with eABR by the ITE MED-EL company device. The cochlear test electrode contains four electrode contacts. It is intended to be inserted into the cochlea during surgery. The length of the electrode is 18 mm, as indicated by the marker ring. Three of the electrode contacts were placed directly into the cochlea (electrodes 1, 2, and 3 corresponding to electrodes number 9, 7, and 5 of a Med-EL Flex28 array, respectively) with the fourth electrode contact placed under the temporalis muscle as ground. A MED-EL technician and a dedicated otoneurologist performed the surgical monitoring with eABR before, during, and after tumor removal. Any modification of the ITE recording was communicated to the surgeon. Responses were also recorded at the end of tumor removal and after CI placement.

ITE and eABR machine were set up as required by the MED-EL instructions and as described by Polak (25). eABR stimulation was performed in monopolar and bipolar mode. The choice to implant every patient with an anatomically preserved nerve despite the eABR response was to assess the predictive value of the test.

### Outcome Evaluation and Data Analysis

eABR after tumor dissection was considered the index test and was categorized according to Walton et al. (26). A score >0 was considered a positive index test result, a score of 0 was considered negative. Wave presence was assessed in real time during surgery by evaluating wave morphology and repeatability, despite their amplitude.

The accuracy of the test was assessed with a comparison between the intraoperative result of eABR and the sound detection arising by CI stimulation, which was defined as the gold standard. The presence of CI response at last follow-up evaluation was considered the reference test to determine a functioning CN and represented a positive outcome of the gold standard test. Patients reporting any auditory response by CI stimulation tests during follow-up were considered “true positive” (TP) if the intraoperative test was also positive, or “false negative” (FN) if the intraoperative test was negative. Patients who did not achieve any sound detection with CI during follow-up were assessed as false positive (FP) or true negative (TN) if they had a positive or a negative intraoperative ITE eABR, respectively (27). The result of intraoperative monitoring was not routinely accessible to follow-up test examiners, who were consequently blind to intraoperative eABR details.

Accuracy, sensitivity, specificity, positive predictive value (PPV), and negative predictive value (NPV) were calculated to determine index test quality (27).

## RESULTS

### Patients’ Characteristics

Since 2012, 37 patients underwent VS resection and simultaneous CI positioning. Since 2020, the ITE was adopted and applied consecutively in the latest 17 CI candidate patients. Data on the intraoperative electrophysiological reports and audiological follow-up were retrieved. All patients had a minimum follow-up of 3 months. In five cases, despite ITE monitoring being performed during tumor resection, the CN was not anatomically preserved at the end of surgery and CI was not positioned. The remaining 12 patients were included in the study.

The median age of the study population was 55.8 years (range, 10–73 yr). Three patients were males (25%) and nine were females (75%). One patient was affected by neurofibromatosis type 2 (8.3%). Seven VSs were intrameatal (58.3%) and five reached the CPA (41.7%) with median extrameatal size of 7 mm (range, 2–13 mm); no case, except one, exceeded the size of 10 mm (13 mm, an exception to the cutoff size of 10 mm because of the patient’s strong will to a CI placement attempt). Extension to the fundus of the internal auditory canal was present in nine cases (75%). Two patients (16.7%) had cystic areas within the tumor.

Median preoperative PTA was 54.4 dB (range, 30–120 dB) ipsilaterally and 16.9 dB (range, 10–42.5 dB) contralaterally. The median WRS was 55% (range, 100–0%) ipsilaterally and 100% (range, 100–50%) contralaterally. One patient with a preoperative 30 dB PTA, 100% WRS, absent ABR and growing VS was unfit for HPS and underwent TL surgery with CI proposal. Conversely, one patient was completely deaf on the tumor side with no WRS detection. Details are reported in Table 1.

**TABLE 1:** Included population features

	Included Population (N = 12)
Age, median (range, yr)	55.8 (10.7–73.2)
Male gender, n (%)	3 (25)
NF2, n (%)	1 (8)
Right sided, n (%)	6 (50)
Intrameatal tumor, n (%)	7 (58)
CPA size, median (range, mm)	7 (2–13)
Free fundal cap, n (%)	2 (17)*^a^*
Preoperative PTA, Ipsi deafness, n (%)	1 (8)
Ipsilateral (median; range, dB)	54.4 (30.0–120.0)
Contralateral (median; range, dB)	16.9 (10.0–42.5)
Follow-up, median (range, mo)	24 (13–45)

*^a^*One case is undetermined.

NF2 indicates neurofibromatosis type 2.

### Surgical Results

Total VS removal was achieved in all patients and no recurrence was observed at last ceMRI follow-up. No major complications or sequalae were observed. The facial nerve was anatomically preserved in every case, with an HB grade I in all cases but one, who was grade II at short-term follow-up.

Of the 17 cases included for intraoperative CN monitoring after tumor removal, CN was anatomically preserved in 12 cases and simultaneous CI was positioned. The adopted criteria to proceed or not with cochlear implantation was anatomical preservation of the nerve.

The CI array was completely inserted in all cases except one, who had two electrodes outside the round window (patient 12); the postoperative CT scan confirmed the intraoperative findings.

### Intraoperative eABR Responses

**FIG. 2:**
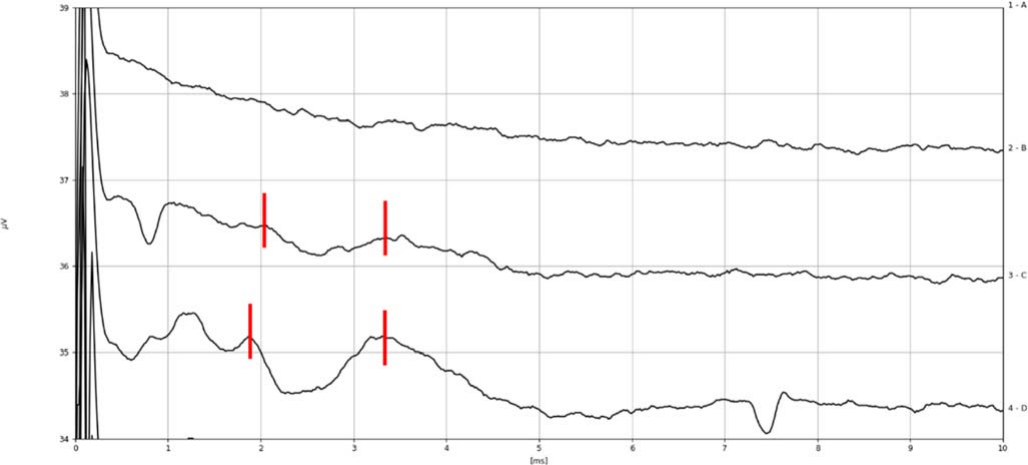
Intraoperative eABR recording of subject 12 before tumor removal with ITE from apical electrode, amplitude 250 cu, phase duration 60 μs (1 μV vertical division, 1 ms horizontal division). Waves eIII and eV are marked in *red*.

**FIG. 3:**
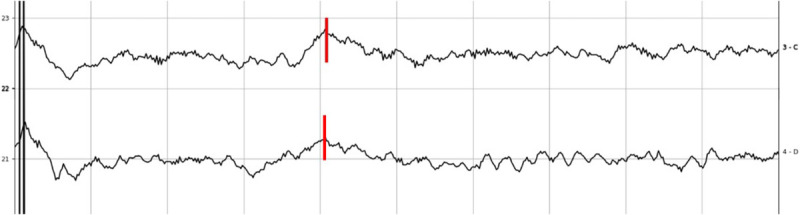
Intraoperative eABR recording of subject 12 during tumor removal with ITE from apical to mid electrode, amplitude 1,000 cu, phase duration 70 μs (*top*), 80 μs (*bottom*) (1 μV vertical division, 1 ms horizontal division). Waves eV are marked in *red*.

**FIG. 4:**
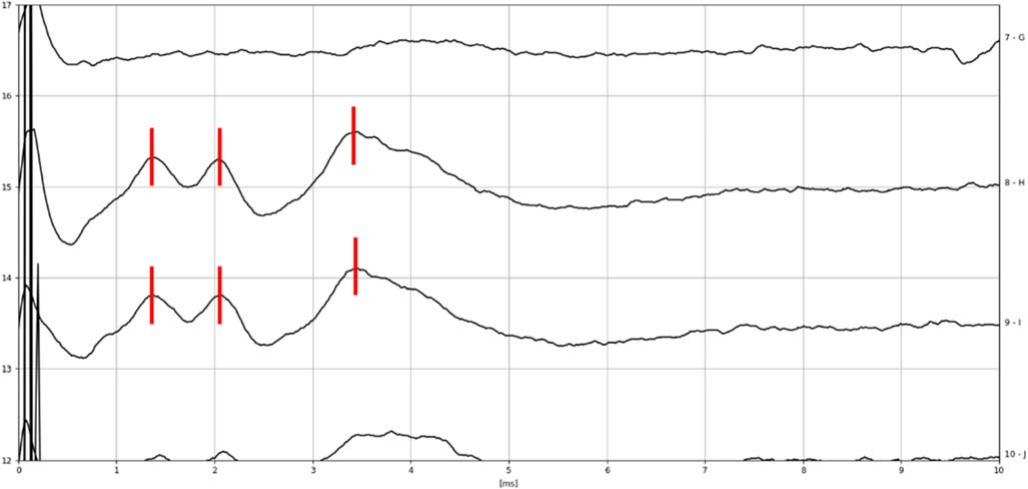
Intraoperative eABR recording of subject 12 after tumor removal with ITE from apical electrode, amplitude 250 cu, phase duration 60 μs (1 μV vertical division, 1 ms horizontal division). Waves eII, eIII, and eV are marked in *red*.

**FIG. 5:**
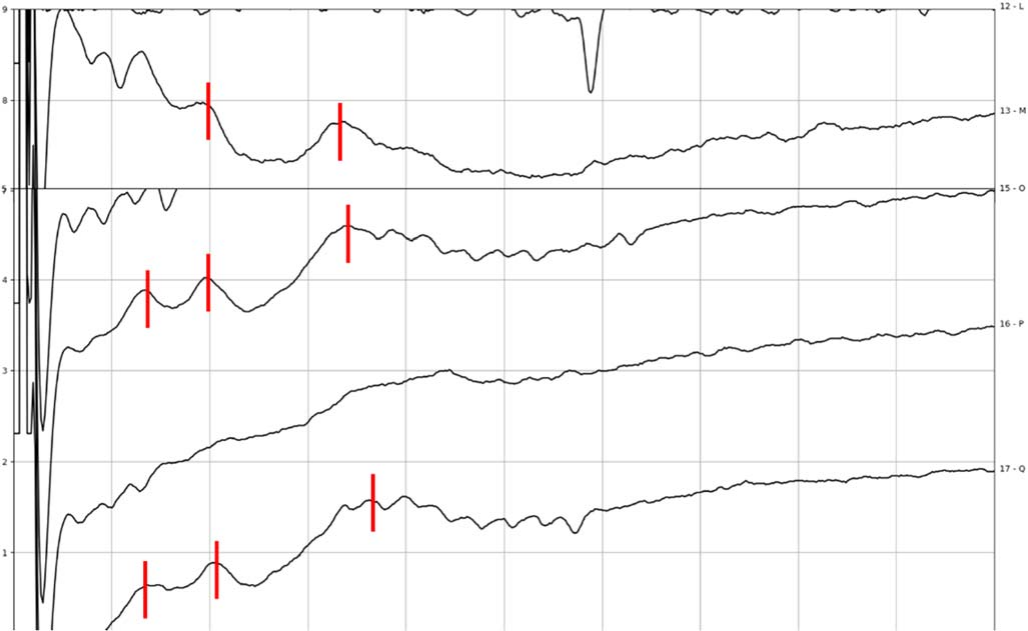
Intraoperative eABR recording of subject 12 with CI from electrode 1 (apical), 300 cu/60 μs, electrode 5 (mid), 500 cu/60 μs, electrode 8 (base) 400 cu, and 500 cu/60 μs (1 μV vertical division, 1 ms horizontal division). Waves eII, eIII, and eV are marked in *red*.

The reliability and predicting power of the test was explored considering the 12 cases where the CN was anatomically intact and CI were placed. eABR with ITE was evaluated in all cases before and after tumor dissection (see Figs. 2–5). Intraoperative positive responses were recorded in 6 of 12 cases (55%) before tumor removal and in 3 of 12 cases (25%) after tumor removal. Soon after CI positioning, the presence of eABR waves was recorded in 3 of 12 cases (25%). Details on eABR intraoperative response for each case are reported in Table 2.

**TABLE 2:** Included patients’ details

Patient	Preoperative PTA (dB)		eABR Results with ITE			eABR Results with CI	Auditory Response by CI Stimulation (GS)	PTA with CI at 6 mo of FU (dB)	PTA with CI at Last FU (dB)	WRS with CI at Last FU	Last FU (mo)	Meaning in ITE Accuracy Test
	Ipsi	Contra	Pretumor Dissection	Posttumor Dissection (IT)	Max Current (Current Unit/Phase Duration [μs], Charge [Qu])							
1	55	33,75	0	0	900/100, 90	0	0	>120	>120	–	45	TN
2	61,25	42,5	0	0	1,000/200, 200	0	0	>120	>120	–	40	TN
3	73,75	10	0	0	700/100, 70	0	1	92,5	79	n.a.	38	FN
4	53,75	31,25	1	1	400/60, 24	0	1	>120	65	20%	34	TP
5	30	10	1	1	900/70, 63	0	1	52,5	35	70%	28	TP
6	>120	13,75	1	0	1,000/200, 200	0	0	>120	>120	–	25	TN
7	53,75	13,75	1	0	800/200, 160	1	1	66,25	45	80%	23	FN
8	78,75	16,25	0	0	600/150, 90	0	0	>120	>120	–	22	TN
9	46,25	13,75	0	0	700/60, 42	0	1	86^a^	68	100%	22	FN
10	48,75	33,75	1	0	1,000/200, 200	0	0	>120	>120	–	16	TN
11	62,50	22,50	0	0	700/60, 42	1	1	50	41	30%	14	FN
12	45	17,5	1	1	1,000/100, 200	1	1	60	37,75	60%	13	TP

*^a^*Values calculated at 500, 1,000, and 2,000 Hz; no response at 4,000 Hz.

FN indicates false negative; FP, false positive; FU, follow-up; GS, gold standard test; IT, index test; n.a., not achieved; TP, true positive; TN, true negative.

### Cochlear Implants Outcome

The median follow-up time was 24 months (range, 13–45 mo); 6 of 12 patients had more than 24 months of follow-up. Despite the duration of follow-up, five cases (42%) did not achieve any sound perception with the CI, even though the CN was anatomically preserved. Among the seven cases with sound perception, the median PTA with CI was 66 dB (range, 50–120 dB) after 6 months of follow-up and 45 dB (range, 35–79 dB) at last evaluation. Patient 4 had no sound perception after 6 months but showed progressively increasing perception during follow-up; at the last evaluation, the PTA was 65 dB and the WRS 20%. Four patients (5, 7, 11, and 12) had <50 dB PTA at last evaluation. Six of seven cases with sound perception also achieved open set speech perception (median WRS, 65%; range, 20–100%), as detailed in Table 2. Mean preoperative PTA was 52 dB in patients who achieved sound perception and 73 dB in those who did not. The eABR with ITE was present in three cases and absent in four of the seven cases who achieved sound perception.

### Data Analysis

According to the categorization parameter reported in the method section, three cases (25%) were accounted as true positive, five cases (42%) as true negative, and four cases (33%) as false negative. No cases were accounted as false positive.

ITE with eABR after tumor resection (the index test), when compared to the perception of sound by CI stimuli (gold standard test), achieved an accuracy of 66.7% (95% CI, 34.9–90.1%). Sensitivity was 42.9% (95% CI, 9.9–81.6%) and specificity 100% (95% CI, 35.9–100%). PPVs and NPVs were 100% (95% CI, 19.4–100%) and 55.6% (95% CI, 21.2–86.3%), respectively (Table 3).

**TABLE 3:** Capability of the eABR with ITE to predict sound detection with cochlear implant

Parameter	Value (%)	95% CI
Accuracy	66.7	34.9–90.1
Sensitivity	42.9	9.9–81.6
Specificity	100	35.9–100
Positive predictive value	100	19.4–100
Negative predictive value	55.6	21.2–86.3

## DISCUSSION

The possibility of predicting CI outcome in VS surgery, in order to maximize the choice of good candidates after tumor removal, is of paramount importance. The issue is relevant when contextual CI implantation is planned after TL surgery, where the surgeon’s judgment of nerve integrity is currently the only available method, despite certain limitations. In fact, despite an anatomically intact CN, 5 out of 12 cases in our study did not achieve any sound perception, highlighting the need for more reliable assessment methods.

Intraoperative monitoring of the CN function through electric stimulation relies on the same principles as stimulating a deaf patient with CI. The premise is the presence of a CN able to receive and propagate electric stimulation.

Surgical preservation of an anatomically intact CN is to be combined with a good quality nerve. CI in VS followed the principle of early surgery: the preservation of an anatomically intact and functioning CN seemed to be related to tumor size and the unforeseeable adherence at the tumor–nerve interface, which, in turn, could hardly be predicted by preoperative hearing. Early surgery favored tumor resection in small tumors, with HPS and sequential CI in case of failures, or with TL surgery and contextual CI (9,28). A successful CI outcome is likely to be expected in cases of HPS failures, where preoperative hearing would allow an easier dissection from the tumor and the preservation of a good quality CN. Conversely, assessing the residual function of the CN after TL approach and tumor removal could be more difficult.

Investigating the role of the intraoperative testing electrode by MED-EL was to assess if it was reliable to select between the cases worth being implanted and the cases which were not, thus reducing the number of potentially unsuccessful implants. At present, few series (17–19) reported their experiences with this tool and pointed out the weaknesses of the false-negative cases that were not systematically investigated.

In our study, the intraoperative electrode test was also applied during tumor dissection (see timeline in Fig. 1) and provided real-time feedback on the state of the nerve through repeated and periodic ITE stimulation.

### Intraoperative Assessment of CN Function and Prediction of Outcome

The ECAP is the measurement of the neural response of spiral ganglion cells to the electrical stimuli provided by the CI. The potential is generated by the most distal part of the auditory pathway, so even if it is easy to measure, it does not reveal lesions proximal to the first neuron (17,29). The eABR evoked by CI stimuli was reported to correlate with CI performance in those patients with positive eABR achieved sound recognition despite a variable degree of speech perception (30).

The Auditory Nerve Test System, certified in 2019 by MED-EL, involved the use of an intracochlear test array (i.e., the ITE) with four electrodes that would be atraumatically inserted through the round window to obtain direct electrical stimulation of the CN. The tool reproduces the electrical stimuli of a CI and acts as a tester to determine if the nerve has any responses (25,31).

This method was proposed in 2017 by Cinar et al. (31) and Lassaletta et al. (16). The first group performed the test in children with malformed cochlea to decide whether patients were suitable for ABI or CI and concluded that if eABR is negative, the results should be evaluated in light of preoperative audiological testing and MRI findings (31). Lassaletta et al. (16) tested deaf patients who were implanted for sensorineural hearing loss: the procedure proved to be reliable and safely correlated with eABR as recorded after CI positioning. Since 2020, it has been applied in VS patients to evaluate its accuracy in predicting CI outcomes (17–19). Despite a PPV of 100% and NPV of 80%, the issue of false-negative cases remained unsolved since the reliability of a negative intraoperative test was still to be verified. In the experience of Medina et al. (17), four cases with intraoperative negative eABR and not sectioned CN were implanted, the other four negative eABR cases did not receive CI. The authors state that “There were eight cases with a negative eABR after tumor resection. If all of them had been implanted, the value of negative predictive value would have risen in all probability.” In Gadenstaetter et al. (19), a similar conclusion was evidenced, addressing the missing point of the false-negative cases and the rate of NPV, which could be higher than investigated. In their paper, it is stated that “Another limiting factor of this study is the missing information on CI outcomes of patients with false-negative eABR due to the applied inclusion criteria. As reported by Medina et al., who also investigated the predictive value of eABR for simultaneous CI in VS surgery, few patients with negative eABR responses after VS removal can still achieve auditory perception after CI.”

Consequently, to date, the issue of false-negative cases raised in the literature has not been solved, as in most cases, only the positive cases were all implanted. It is known, as happened in our experience too, that the nerve may be suffering immediately after tumor removal. In two of our cases, a negative eABR recorded at the end of surgery, typically coinciding with the completion of VS removal, turned positive after a few minutes during the surgeons’ final inspection of the CPA before CI positioning. We considered that, from a methodological point of view, implanting all cases with a preserved CN, regardless of the intraoperative presence or absence of eABR, was the most effective approach to investigate the occurrence of false-negative cases.

### CI Outcomes and Open Issues

The audiological follow-up of our patients revealed sound detection in 7 of 12 cases and some degree of speech discrimination in 6 of 12 (median WRS, 65%; range, 20–100%). The present paper was not centered on CI outcomes, which were reported for completeness, since the follow-up period needed for a reasonable assessment of results after VS resection should be at least 2 years (32). In our series, all cases had a minimum follow-up of 1 year, but only 50% reached 2 years. Previous and more complete data on our overall experience of CI after VS resection was published (32) and only the cases with 2 or more years of follow-up were considered, including both sporadic and NF2 cases.

Comparison among different experiences, as mentioned in the literature (33), is scarcely feasible because of the heterogeneous methods of selecting patients, measuring, and reporting outcomes (32,33). No agreement was achieved in the literature on the methods to explore the efficacy of CI after VS resection or on the masking noise to adopt in contralateral hearing when present. In Sanna et al. (34), CI rehabilitation after VS resection in single-sided deafness conditions was explored using 40 dB masking noise, making it difficult to compare with other experiences where higher (or lower) levels of masking noise were applied to the only hearing ear. In our preliminary series of CI after VS resection (32), we investigated several prognostic factors: preoperative hearing and the condition of the contralateral hearing were the most relevant, positively and negatively influencing CI outcomes, respectively. In West et al. (33), poor contralateral hearing was retrieved as a positive prognostic factor for CI success and was present in approximately 50% of the patients. In addition, this paper also did not provide any information about the masking strategy. Similarly, in Lassaletta et al. (35), 6 of 15 cases had poor contralateral hearing (>50 PTA and <50% WRS). Tadokoro et al. (36) reported a median WRS with CI of 46% for NF2 patients and 60% for sporadic VS patients, with a mean follow-up of 39 months and no data on masking strategy.

In light of the good contralateral hearing in most patients in our series, with short follow-up available for half of them, the present CI hearing outcomes should be considered preliminary.

### Our Results with ITE

Patients who intraoperatively reported any response at electric stimulation were considered positive results, which meant that the CN was able to conduct a stimulus and would, in principle, allow the candidacy to CI. Response to CI stimulus (sound detection) was the chosen gold standard.

The results of our series, as well as the CI audiological outcomes, were influenced by the inclusion criteria, which considered for TL surgery only those cases with small growing tumor and poor ipsilateral preoperative hearing, or when there were no favorable conditions for HPS. Poor hearing and the long-standing presence of tumor are supposed to represent a condition of CN damage, which could lead to less satisfying results with CI and likely higher failure rate. The only case with 30 dB preoperative PTA had a growing tumor but overall unfavorable conditions to HPS and was agreed to be treated with TL surgery and CI. The postoperative CI outcome was 35 dB PTA.

Five out of seventeen cases enrolled for ANTS testing did not achieve nerve preservation and were excluded. The case with preoperatively ipsilateral deafness (patient 6) showed no CI response at 25 months follow-up.

In this study, the intraoperative eABR evaluation with ITE achieved an accuracy of 66.7%, a sensitivity of 42.9%, and a specificity of 100%. These data mean that, in our series, 43% of patients able to perceive sound with their CI (positive gold standard) displayed the presence of waves at the intraoperative eABR (positive ITE after tumor removal before CI placement), while all patients unable to perceive any sound (negative gold standard) with their CI displayed intraoperative negative eABR (negative ITE after tumor removal before CI placement). Conversely, out of nine intraoperative negative case, four showed response to CI. When the intraoperative testing was compared with the CI outcome, 4 (33.3%) of 12 were false-negative cases whereas no cases of false positives were found.

PPVs and NPVs were 100% and 55.6%, respectively: this pointed to the fact that a patient with a positive intraoperative eABR had a 100% probability of obtaining sound perception with CI, but negative eABR could not rule out that CI would work.

These values seem to suggest that, when a positive eABR is intraoperatively clearly recorded, ITE was a viable option to support the surgeon’s judgment on the functional status of the CN and successful CI positioning but, when no action potential is detected, ITE did not rule out whether the CI would give any response and its role in selecting candidates for CI is still to be defined. These data can be elucidated by the fact that the detection of an action potential requires the simultaneous activation of multiple nerve fibers to generate a clearly detectable potential. In contrast, the perception of hearing may be the result of the activation of a smaller number of fibers. Regarding VS, it is important to note that the action potential is measured immediately after surgery. Therefore, surgical trauma, reduction of CN fibers, exposure of the nerve, and blood supply reduction (even temporarily) may impact the recording even though vital fibers are still present.

Our study revealed a higher percentage of false negatives compared to previous studies, which are, however, difficult to compare with the present one due to differing methodologies, as we discussed. Nonetheless, this might represent a limitation of our study. We presented the results as they were recorded and interpreted by experts (neurophysiologists and technicians) in the operating room. The difficulties in interpreting the responses, the long learning curve required to gain experience, as well as the rarity of cases, should be taken into consideration. The results may also be contaminated by electrical artifacts and/or myogenic stimulation, potentially mimicking or masking the auditory response, as seen in waves eIII and eV. A weak and small amplitude or increased latency of nerve response after manipulation for tumor removal might also be misjudged. A retrospective and blind reappraisal of all traces, once the CI is activated, might provide different results and give a partial explanation of the false-negative cases. The setting of the operating room is complex and decisions on CI positioning are to be taken soon after tumor removal. At present, our conclusions are based on real-time intraoperative evaluation of traces, which may not be as accurate as evaluations conducted in an office setting. Additionally, the occurrence of cases without a positive eABR even before tumor resection—despite relatively good preoperative hearing (e.g., case 9 in the present study or case 20 in Medina et al. (17)—suggests possible technical limitations of the test itself.

Thus far, all the mentioned intrinsic limitations may contribute to the wide variability in the number of false negatives. This issue has been raised in the literature (17,19) and the design of our study was specifically intended to address it, i.e., by implanting all patients regardless of the intraoperative ITE response. This makes it difficult to compare the accuracy of our test with the other available studies. In Medina et al. (17), 93% accuracy, 90% sensitivity, 100% specificity, 100% PPV, and 80% NPV were reported. The preliminary study on ITE in inner ear malformations by Cinar et al. (31) reported only one case (10%) with a false negative. In other studies, only positive eABR cases were implanted, whereas in others (17) some of the negative cases were implanted and others were not. In all these studies, it was questioned whether the potential rates of false negatives would be higher.

To date, this is the first study to retrospectively investigate the accuracy of ITE in a case series where, once the nerve was preserved after tumor removal, CI was placed despite the result of the intraoperative eABR. This choice aimed to reduce bias in the evaluation of test accuracy and aimed to assess its the predictive value. Further evaluation through blind interrater testing will promote experiences in the interpretation of data and might finally lead to a revision of ITE accuracy parameters and perhaps enhance the reliability of the test. Similarly, longer follow-up and clinical outcome assessment will represent a more reliable parameter of judgment (32). At present, short follow-up represents a limitation since we do not know if positive response to our gold standard test entails a good CI performance. Further clinical data will be provided to clarify the role of this intraoperative test in predicting CI outcome.

## CONCLUSION

The CI was placed in 12 patients whose CN was intact after tumor removal; seven achieved sound detection. Our analysis showed that when eABR was intraoperatively evoked after tumor resection, CI placement was likely to be successful. The cases that, despite no intraoperative response, showed an audiological response pointed out the risk of the false-negative test. Beside negative ITE, other factors like the quality of the preserved nerve should be weighted to better understand mechanisms of success/unsuccess and the interactions of different factors in VS surgery and CI placement. The reliability of the intraoperative testing is to predict and reduce unsuccessful cases but, at the same time, not prevent from rehabilitation those cases who would benefit from CI.

This preliminary study outlines that, despite the high positive predictive value of MED-EL ITE, further studies, in-depth analyses of traces, and longer follow-up are necessary to analyze its role. At present, false-negative cases lead to the conclusion that not only the intraoperative response of eABR but also the anatomical preservation of the nerve, along with other clinical factors such as preoperative ipsilateral and contralateral hearing and NF2 status, should be considered collectively to determine whether a cochlear implant is appropriate in each individual case. Longer follow-up with multicentric studies and multiple raters’ evaluations of eABR traces will be likely to clarify the issue and confirm the accuracy of the ITE.
